# Folate metabolism in man: the effect of malignant disease.

**DOI:** 10.1038/bjc.1982.210

**Published:** 1982-09

**Authors:** A. M. Saleh, A. E. Pheasant, J. A. Blair, R. N. Allan, J. Walters

## Abstract

The metabolism of [2-14C]+[3', 5', 7, 9-3H] folic acid and [214C]+[3', 5', 7, 9-3H] 10-formylfolate was studied in hospital inpatients. Metabolites detected in the urine after folic acid feeding included the unchanged compound, other folates and a number of breakdown products, such as p-acetamidobenzoyl-L-glutamate and p-acetamidobenzoate. This confirms the existence of a folate catabolic pathway in man. Patients with malignant disease excreted less of the dose in urine, incorporated more into the reduced folate pool, and showed decreased catabolism of folate, when compared to controls. 10-Formylfolate was excreted largely unchanged, and appears not to be reduced by man. Also 10-formylfolate interfered with the reduction of folic acid given simultaneously.


*Br. J. Cancer* (1982) **46**, 346

### FOLATE METABOLISM IN MAN: THE EFFECT OF MALIGNANT DISEASE

A. M. SALEH*, A. E. PHEASANT*, J. A. BLAIR*, R. N. ALLAN† AND J. WALTERS‡

*From the *Department of Chemistry, University of Aston in Birmingham, Birmingham B4 7ET*
*† The General Hospital, Steelhouse Lane, Birmingham B4 6NH*
*and the ‡Department of Gastroenterology, SUNYAB, Erie County Medical Centre, Buffalo NY 14215, U.S.A.*

Received 4 January 1982 Accepted 20 April 1982

**Summary.—The metabolism of [2-$^{14}$C]+[3′, 5′, 7, 9-$^{3}$H] folic acid and [2$^{14}$C]+[3′, 5′, 7, 9-$^{3}$H] 10-formylfolate was studied in hospital inpatients. Metabolites detected in the urine after folic acid feeding included the unchanged compound, other folates and a number of breakdown products, such as p-acetamidobenzoyl-L-glutamate and p-acetamidobenzoate. This confirms the existence of a folate catabolic pathway in man.**

**Patients with malignant disease excreted less of the dose in urine, incorporated more into the reduced folate pool, and showed decreased catabolism of folate, when compared to controls. 10-Formylfolate was excreted largely unchanged, and appears not to be reduced by man. Also 10-formylfolate interfered with the reduction of folic acid given simultanteously.**

EARLIER STUDIES on the metabolism of folic acid by man, using $^{3}$H-labelled tracers (Chanarin & McLean, 1967) or microbiological assay (Ratanasthien, 1975), yielded conflicting results. There have been no detailed studies since the introduction of the unambiguous technique of using a mixture of $^{14}$C- and $^{3}$H-labelled species by ourselves, and the recognition of a breakdown process as a major route of metabolism of folates in the rat (Murphy *et al.*, 1976; Connor *et al.*, 1979).

It is believed that two pools of folate exist in the rat; a short-term pool of folate monoglutamates giving p-acetamidobenzoate as a urinary catabolite, and a long-term pool of folate polyglutamates giving p-acetamidobenzoyl-L-glutamate (Pheasant *et al.*, 1981). The identification of $^{14}$C-labelled pterins in the urine of one human subject given $^{14}$C-folic acid (Krumdieck *et al.*, 1978) and $^{3}$H-p-acetamidobenzoyl-L-glutamate in the urine of a limited number of subjects given $^{3}$H- +$^{14}$C-labelled folic acid (Pheasant *et al.*, 1979), together with the pattern of excretion of radioactivity in these experiments, suggest that the same metabolic pools and catabolic routes may exist in man.

Folates are essential for cell division, and folate status is therefore important in malignant disease. Folate deficiency is associated with malignant disease (Blakley, 1969; Chanarin, 1979) but no detailed comparative metabolic studies have been carried out in man. Catabolism of folate in the rat has recently been shown to be reduced in the presence of a tumour (Saleh *et al.*, 1981). Therefore the metabolism of a mixture of $^{14}$C- and $^{3}$H-labelled folic acid was studied in control patients and in patients with malignant disease in order to observe any metabolic variations which may have diagnostic or therapeutic applications. Preliminary results have been reported elsewhere (Saleh *et al.*, 1980).

10-Formylfolate, an oxidized folate, is an important constituent of food folate (Butterworth *et al.*, 1965; Santini *et al.*,

1964). However, it is not reduced by Ehlrich ascites carcinoma (Bertino *et al.*, 1965) or bovine dihydrofolate reductase (A. Sahota, personal communication) and therefore may not be available to mammals. Earlier investigations by Ratanasthien *et al.* (1974), using a microbiological assay, showed no metabolism of orally administered 10-formylfolate by man. However, Pheasant *et al.* (1981) found that radiolabelled 10-formylfolate was incorporated into the reduced folate pool of the rat. Further studies undertaken using radiolabelled 10-formylfolate in man, to resolve these apparent discrepancies, are described here.

### MATERIALS AND METHODS

### *Experimental design*

*Patients.*—The study was carried out on hospital inpatients (General Hospital, Birmingham) suffering from malignant disease or other disorders. Details of the patients may be found in Table I. All patients were allowed a normal diet throughout the experiment. Informed consent was obtained from all participants.

*Metabolism of folic acid.*—Two groups of patients were used: Group I—11 patients suffering from malignant disease; Group II.—6 patients suffering from other disorders as controls. All patients were given an oral dose of a mixture of 5 $\mu$Ci [2-$^{14}$C] folic acid and 20 $\mu$Ci [3′, 5′, 7, 9-$^{3}$H] folic acid plus 5 mg unlabelled folic acid.

*Metabolism of 10-formylfolic acid and its effect on folic acid metabolism.*—Three groups of 3 control patients were used:

Group III.—Patients were given a mixture of [2-$^{14}$C] folic acid + [3′, 5′, 7, 9-$^{3}$H] folic acid (60 $\mu$g).

Group IV.—Patients were given a mixture of [2-$^{14}$C] + [3′, 5′, 7, 9-$^{3}$H] 10-formylfolic acid + 5 mg unlabelled 10-formylfolic acid.

Group V.—Patients were given a mixture of [2-$^{14}$C] + [3′, 5′, 7, 9-$^{3}$H] folic acid (60 $\mu$g) + 5 mg unlabelled 10-formylfolic acid.

### *Urine and faeces collection*

Urine was collected on sodium ascorbate (10 g) for the following periods; 0–6 h, 6–12 h and 12–24 h after administration of the dose. Urine volumes were measured for each period, and samples kept frozen at −20°C until analysis, which was within a week of collection. In some cases faeces were collected in a plastic bag, which was closed with a rubber band and put into an air-tight container.

### *Determination of radioactivity*

Urine samples and column effluents were counted as described in Connor *et al.* (1979). Faeces were freeze-dried and ground to give a homogeneous powder; 100mg samples were used to estimate total radioactivity as described by Barford *et al* (1978).

### *Chromatography*

Sephadex G15 gel filtration and DEAE–cellulose chromatography (using linear gradients of 0–1·2M NaCl in 0·05M sodium phosphate buffer, pH 7·0) were performed as described previously (Barford *et al.*, 1977). Paper chromatography was performed as described by Connor *et al.* (1979).

### *Chemicals*

All chemicals used were of Analar grade or its equivalent. [2-$^{14}$C] Folic acid (sp. 50 mCi/mmol) and (3′, 5′, 7, 9-$^{3}$H] folic acid (sp. 500 mCi/mmol) were obtained from the Radiochemical Centre, Amersham, Bucks. p-Acetamidobenzoyl-L-glutamate was prepared as described in Baker *et al.* (1964). 10-Formylfolic acid was synthesized from folic acid (Blakley, 1959), and [2-$^{14}$C] and [3′, 5′, 7, 9-$^{3}$H) 10-formylfolic acid was prepared as follows: a solution of [2-$^{14}$C] folic acid (50 $\mu$Ci, 52·4 mCi/mmol) and [3′, 5′, 7, 9-$^{3}$H] folic acid (250 $\mu$Ci, 500 mCi/mmol) was dissolved in 0·8 ml of distilled formic acid (98%). After storage of the solution in the dark at room temperature for 48 h, the excess formic acid was removed by freeze-drying. Chromatography of the product on DEAE–cellulose gave a single radioactive peak at 0·53M NaCl, co-chromatographing with authentic 10-formylate (folic acid elutes at 0·96M NaCl).

### RESULTS

### *Metabolism of folic acid*

The urinary recoveries of radioactivity after an oral dose of labelled folic acid (5 mg) are shown in Table II. There is a discrepancy between the recovery of $^{3}$H and $^{14}$C in the urine. Urinary recovery of $^{3}$H was significantly higher than $^{14}$C in

TABLE I.—*Clinical details of patients studied*

| Group | Name | Age | Sex | Diagnosis | Therapy |
|---|---|---|---|---|---|
| I | N.C. | 48 | M | Cancer of oesophagus | None |
| | G.R. | 60 | M | Lymphosarcoma | Daunarubicin, Ara-C thioguanine |
| | B.B. | 63 | F | Adenocarcinoma of ascending colon | None |
| | K.M. | 68 | F | Bronchial carcinoma and metastases in thoracic spines | Cyclizine-HCl, dihydrocodeine, Distalgesic |
| | H.P. | 67 | M | Bronchial carcinoma | None |
| | I.G. | 75 | F | Cancer of breast—diabetes | Morphine, Tamoxophen, chlorpropamide |
| | C.H. | 68 | M | Cancer of lung | Radiotherapy |
| | L.J. | 64 | M | Lymphosarcoma | Radiotherapy, paracetamol |
| | J.H. | 73 | M | Adenocarcinoma of the stomach | None |
| | D.M. | 17 | M | Lymphocytic lymphoma | Prednisone, K supplements |
| | W.M. | 66 | M | Non-Hodgkin's lymphoma | Prednisone, pethidine |
| II | C.C. | 66 | M | Chronic bronchitis and emphysema | Salbutamol, diuretics |
| | N.R. | 67 | M | Hypertension | Diuretics |
| | S.R. | 74 | M | Myocardial infarction | Diuretics |
| | E.W. | 68 | F | Cervical spondylitis | Dihydrocodeine |
| | E.T. | 82 | M | Deep-vein thromosis | Digoxin and diuretics |
| | L.W. | 55 | M | Hypertension—cerebrovascular accident | None |
| III | A.G. | 59 | M | Myocardial infarction | Moduretic |
| | P.H. | 63 | M | Hypertension and possible myocardial infarction | Thiazide—Distalgesic Triazolam |
| | T.C. | 56 | M | Anterolateral myocardial infarction | None |
| IV | R.S. | 57 | M | Myocardial infarction | Nitrazepam |
| | G.L. | 75 | M | Myocardial infarction | Triazolam—aspirin |
| | A.M. | 48 | M | Retroperitoneal fibrosis | Paracetamol |
| V | F.C. | 67 | F | Myocardial infarction | Frusemide—Slow K—triazolam |
| | S.H. | 72 | M | Bulbar palsy | None |
| | M.F. | 70 | F | Myocardial infarction—hypertension | Moduretic |

TABLE II.—*Urinary recovery of radioactivity after an oral dose of* [2-$^{14}C$] *and* [3′, 5′, 7, 9-$^{3}H$] *folic acid* (5 *mg*). *The results are expressed as the percentage of the dose recovered during the* 3 *collection periods*

| | % of dose recovered | | | | | | | | |
|---|---|---|---|---|---|---|---|---|---|
| | 0–6 h | | 6–12 h | | 12–24 h | | 0–24 h | | |
| Patients | $^{3}H$ | $^{14}C$ | $^{3}H$ | $^{14}C$ | $^{3}H$ | $^{14}C$ | $^{3}H$ | $^{14}C$ | |
| Cancer | | | | | | | | | |
| N.C. | 23·8 | 20·4 | 4·6 | 3·7 | 1·6 | 0·2 | 30·0 | 24·3 | |
| G.R. | 15·1 | 14·6 | 8·4 | 8·0 | 2·1 | 2·2 | 25·6 | 24·8 | |
| B.B. | 0·3 | 0·2 | 15·0 | 12·0 | 7·0 | 6·0 | 22·3 | 18·3 | Increasing |
| K.M. | 12·3 | 10·4 | 7·0 | 5·9 | 2·4 | 1·7 | 21·7 | 18·0 | tumour mass |
| H.P. | 14·7 | 13·2 | 3·6 | 3·1 | 0·9 | 0·5 | 19·2 | 16·8 | |
| I.G. | 10·2 | 8·0 | | | | | | | |
| C.H. | 1·2 | 0·9 | 8·2 | 7·3 | Died | | | | |
| L.J. | 2·5 | 2·4 | — | — | 2·4 | 0·8 | 4·9 | 3·2 | |
| J.H. | — | — | 1·7 | 1·4 | 3·0 | 1·4 | 4·7 | 2·8 | |
| D.M. | 2·0 | 1·8 | — | — | 0·4 | 0·5 | 2·4 | 2·3 | |
| W.M. | 0·1 | 0·1 | 0·7 | 0·7 | 0·3 | 0·3 | 1·1 | 1·1 | ↓ |
| | | | | | | | 14·1 ± 3·7* | 12·4 ± 3·3* | |
| Control | | | | | | | | | |
| C.C. | 29·0 | 23·3 | 9·0 | 7·4 | 1·2 | 1·1 | 39·2 | 31·8 | |
| N.R. | 8·6 | 8·4 | 24·0 | 22·2 | 4·7 | 3·1 | 37·3 | 33·7 | |
| S.R. | 19·6 | 17·3 | 5·3 | 4·6 | 8·4 | 7·3 | 33·3 | 29·2 | |
| E.W. | 16·5 | 12·3 | 6·6 | 4·9 | 2·9 | 1·6 | 26·0 | 18·8 | |
| L.W. | 12·7 | 11·3 | | | | | | | |
| E.T. | 11·6 | 9·4 | 7·1 | 5·7 | 5·6 | 2·3 | 24·3 | 17·4 | |
| | | | | | | | 32 ± 3·0* | 26·2 ± 3·4* | |

* Mean ± s.e.

TABLE III.—*Faecal recovery of radioactivity after an oral dose of [2-$^{14}$C] and [3′,5′, 7, 9-$^{3}$H] folic acid. The results are expressed as the percentage of the dose recovered in 46 h*

| Patient | Faecal dry weight (g) | %Dose recovered $^{3}$H | $^{14}$C |
|---|---|---|---|
| Cancer | | | |
| L.J. | 87·3 | 10·8 | 28·0 |
| G.R. | 60·0 | 6·7 | 13·9 |
| Control | | | |
| E.W. | 38·1 | 10·3 | 26·6 |
| E.T. | 4·9 | 0·2 | 0·3 |

both groups of patients, when determined by a paired $t$ test ($P < 0{\cdot}001$). Cancer patients excreted significantly less radioactivity in urine than control patients ($P < 0{\cdot}001$); 14·1% $^{3}$H, 12·4% $^{14}$C and 32·0% $^{3}$H and 26·2% $^{14}$C of the dose being excreted in the urine of cancer patients and control patients, respectively. Most of the radioactivity appearing in the urine was excreted in the first 12 h by both groups of patients. Excreted radioactivity fell rapidly on the second day, but was detected at low levels in the urine up to 5 days. The urinary recovery of the individual cancer patients showed an inverse correlation between total urinary recovery and size of tumour. The urinary recovery decreased as the approximate size and extent of the tumour mass, as judged by the clinician, increased, indicating a larger requirement for folate in malignant disease.

Table III shows the recovery of $^{3}$H and $^{14}$C in the faeces; where more $^{14}$C than $^{3}$H was present. No evidence for malabsorption of folate in malignant disease was apparent from these investigations.

### *Urinary metabolites*

Urine samples were sequentially chromatographed on DEAE-cellulose, Sephadex G15 and paper. In all cases, this revealed a number of radioactive components. Three of the components detected in the urine retained the $^{3}$H- and $^{14}$C-label in the same ratio, and were identified by co-chromatography with authentic standards in both column systems as folic acid, 5-methyl-

TABLE IV.—*Metabolites present in the urine collected 0–24 h after the feeding* [2-$^{14}$C] [3′, 5′, 7, 9-$^{3}$H] *folic acid. The results are expressed as the percentage of the dose as each metabolite.*

| | % dose | | | | | |
|---|---|---|---|---|---|---|
| | Folic acid | | 5MeTHF | | pAcBG | pAcBA |
| Patient | $^{3}$H | $^{14}$C | $^{3}$H | $^{14}$C | $^{3}$H | $^{3}$H |
| Cancer | | | | | | |
| N.C. | 18·8 | 17·0 | 3·8 | 3·7 | 0·9 | 0·1 |
| G.R. | 8·9 | 9·2 | 4·7 | 5·3 | 2·8 | 0·1 |
| B.B. | 11·6 | 9·5 | 5·3 | 4·9 | 2·7 | — |
| K.M. | 14·1 | 12·8 | 2·0 | 2·1 | 2·6 | 0·3 |
| H.P. | 13·1 | 12·2 | 2·2 | 1·9 | 1·0 | 0·3 |
| C.H. | 5·8 | 5·9 | 1·1 | 1·0 | 1·6 | — |
| L.J. | 1·1 | 1·1 | 0·7 | 0·7 | 1·4 | 1·6 |
| J.H. | 0·1 | 0·1 | 0·2 | 0·2 | 1·6 | 2·2 |
| D.M. | 0·7 | 0·6 | 1·4 | 1·3 | — | — |
| Mean ± s.e. | 8·2±2·2 | 7·6±2·0 | 2·4±0·6 | 2·3±0·6 | 1·6±0·3 | 0·5±0·3 |
| Control | | | | | | |
| C.C. | 26·3 | 20·2 | 5·0 | 4·2 | 5 | — |
| N.R. | 26·2 | 23·3 | 3·3 | 3·3 | n.d. | n.d. |
| S.R. | 22·1 | 20·0 | 5·7 | 5·5 | 3·2 | 0·9 |
| E.W. | 18·2 | 14·1 | 3·6 | 2·7 | 2·2 | 1·4 |
| E.T. | 13·4 | 11·2 | 4·8 | 4·0 | 3·6 | 0·8 |
| Mean ± s.e. | 21·2±2·5 | 17·7±2·2 | 4·5±0·4 | 3·9±0·5 | 3·5±0·6 | 0·8±0·3 |

5MeTHF; 5-methyltetrahydrofolate. pAcBG; p-acetamidobenzoyl-L-glutamate
pAcBA; p-acetamidobenzoate.
n.d.; not determined.

tetrahydrofolate (5MeTHF) and 10-formylfolate. The ratio $^{3}H/^{14}C$ in these folate derivatives was higher than in the folic acid administered. The remaining components detected in the urine of both groups were catabolites, labelled solely with $^{3}H$ or mainly with $^{14}C$. The $^{3}H$-catabolites were identified as p-acetamidobenzoyl-L-glutamate, p-acetamidobenzoate and water. The $^{14}C$-labelled catabolite has not been identified. It eluted from both columns in the position of metabolite B which has been recently reorted in rat urine (Saleh *et al.*, 1981). Table IV summarizes the relative distribution of the major metabolites appearing in the various urine samples of both groups of patients. Cancer patients excreted significantly less unchanged folic acid than control patients ($0{\cdot}01 < P < 0{\cdot}05$). There is no significant difference in 5MeTHF excretion between the two groups, but the ratio of folic acid to 5MeTHF decreased considerably with time in cancer patients, whereas it decreased only slightly in control patients (folic acid/5MeTHF for 0–6 h, 6–12 h, and 12–24 h; 5·2, 3·1, 1·0 for Group I; 5·6, 5,1, 2·8 for Group II). Urinary scission product excretion relative to intact folates increased with time in both groups. However, total scission products were significantly decreased in cancer patients ($0{\cdot}01 < P < 0{\cdot}05$). p-Acetamidobenzoate was not present in 0–6h urine samples of either group and appeared in the 6–12h urine sample of most cancer patients but only one control patient (E.W.). Its excretion was maximal in the 12–24h urine samples of both groups. p-Acetamidobenzolyl-L-glutamate showed a reciprocal pattern: it was maximal in the first urine samples (0–6 h) and decreased slowly as a percentage of the dose thereafter. Overall, p-acetamidobenzoyl-L-glutamate excretion was significantly depressed in cancer patients ($P < 0{\cdot}05$), whereas p-acetamidobenzoate excretion was unchanged.

*Metabolism of* 10-*formylfolic acid and its effect on folic acid metabolism*

*Group III: Metabolism of folic acid* (60 μg).—2·6% of $^{3}H$ and 1·8% of $^{14}C$ of the dose were excreted in the urine in 24 h by control subjects receiving 60 μg folic acid (Table V), and 1·7% $^{14}C$ and 1·3% of $^{3}H$ were recovered in their faeces in 48 h. Thus at this lower dose of folic acid a much

TABLE V.—*Urinary recovery of radioactivity after oral doses of folates.*

| Patient | 0–6 h $^{3}H$ | 0–6 h $^{14}C$ | 6–12 h $^{3}H$ | 6–12 h $^{14}C$ | 12–24 h $^{3}H$ | 12–24 h $^{14}C$ | 0–24 h $^{3}H$ | 0–24 h $^{14}C$ |
|---|---|---|---|---|---|---|---|---|
| Group III | | | | | | | | |
| A.G. | 1·0 | 0·6 | 0·9 | 0·6 | 0·3 | 0·2 | 2·2 | 1·4 |
| P.H. | 1·8 | 1·1 | 0·6 | 0·5 | 0·7 | 0·5 | 3·1 | 2·1 |
| T.C. | 0·8 | 0·6 | 1·2 | 0·7 | 0·5 | 0·5 | 2·5 | 1·8 |
| Mean | | | | | | | 2·6 | 1·8 |
| Group IV | | | | | | | | |
| R.S. | 46·1 | 41·1 | 4·3 | 4·0 | 1·4 | 0·7 | 51·8 | 45·8 |
| A.M. | 40·1 | 38·2 | 5·8 | 5·7 | 0·4 | 0·7 | 46·3 | 44·6 |
| G.L. | 25·4 | 22·4 died | | | | | | |
| Mean | | | | | | | 49·1 | 45·2 |
| Group V | | | | | | | | |
| F.C. | 5·4 | 5·5 | 3·2 | 2·9 | 1·2 | 0·2 | 9·8 | 8·6 |
| S.H. | 23·1 | 22·4 | 5·8 | 5·8 | 1·8 | 1·2 | 30·7 | 29·4 |
| M.F. | 7·6 | 7·5 | 5·0 | 4·6 | n.d. | n.d. | 12·6 | 12·1 |
| Mean | | | | | | | 17·7 | 16·7 |

Group III: [2-$^{14}C$]+[3′,5′,7,9-$^{3}H$]-folic acid (60 μg).
Group IV: [2-$^{14}C$]+[3′,5′,7,9-$^{3}H$]-10 formylfolic acid (5 mg).
Group V: [2-$^{14}C$]+[3′,5′,7,9-$^{3}H$]-folic acid (60 μg)+5 mg unlabelled 10 formylfolic acid.
The results are expressed as the percentage of the dose recovered in the different collection periods.
n.d.: not determined.

TABLE VI.—*Metabolites present in the urine collected 12 h after the administration of labelled folates*

| Patient | Folic acid | | 5MeTHF | | 10-formylfolic acid | |
|---|---|---|---|---|---|---|
| | $^3H$ | $^{14}C$ | $^3H$ | $^{14}C$ | $^3H$ | $^{14}C$ |
| Group IV* | | | | | | |
| R.R. | 1·5 | 1·4 | 0·9 | 0·9 | 46·4 | 41·3 |
| A.M. | 2·1 | 2·1 | 1·1 | 1·2 | 40·4 | 39·0 |
| G.L.† | 0·9 | 0·8 | 0·5 | 0·4 | 23·7 | 20·7 |
| Group V* | | | | | | |
| M.F. | 10·0 | 9·9 | 0·9 | 0·9 | 0·5 | 0·5 |
| S.H. | 23·8 | 23·2 | 2·3 | 2·3 | 0·6 | 0·7 |
| F.C. | 6·2 | 6·5 | 0·9 | 0·9 | 0·5 | 0·5 |
| Mean | 13·3 | 13·2 | 1·4 | 1·4 | 0·6 | 0·6 |

The results are expressed as the percentage of the dose present as each metabolite.
* As in Table V.
† 0–6 h only.

greater proportion of the dose was retained in the body than in the 5mg dose. Chromatographic analysis of the urine samples showed that radioactivity in the urine from subjects given the low dose was present only as scission products. No folic acid was detected in the urine and little or no 5MeTHF. The major urinary catabolite was labelled only with $^3H$ and identified as p-acetamidobenzoyl-L-glutamate. p-Acetamidobenzoate was also detected. The $^{14}C$-labelled species was different from that found earlier, and remains unidentified.

*Group IV: Metabolism of* 10-*formylfolic acid.*—The urinary recovery of the radioactivity in 24 h after the administration of [2-$^{14}C$]+[3′,5′,7,9-$^3H$] 10-formylfolic acid is given in Table V. 49·1% of $^3H$ and 45·2% of $^{14}C$ of the dose were recovered in 24 h in the urine. Excretion of radioactivity in the urine was maximal in the first period and dropped sharply thereafter. Urine samples were sequentially chromatographed on DEAE–cellulose and Sephadex G15, and this revealed a number of radioactive components. More than 90% of the urinary radioactivity was identified as 10-formylfolic acid. The other metabolites detected in the urine were folic acid, 5-MeTHF and a very small amount of $^3H$-labelled catabolites. More folic acid was excreted than 5MeTHF, but the total amount of both did not exceed 3% of the dose (Table VI).

*Group V: Effect of* 10-*formylfolic acid on folic acid metabolism.*—Subjects receiving oral doses of a mixture of [2-$^{14}C$]+[3′,5′,7,9-$^3H$] folic acid (60 $\mu$g)+5 mg unlabelled 10-formylfolic acid excreted in their urine 17·7% $_3H$ and 16·7% $^{14}C$ of the labelled dose (Table V). The recovery of $^3H$ and $^{14}C$, in individual samples were quite similar, particularly in the 0–6 and 6–12h urine samples. Table VI shows the distribution of radiolabelled folate derivatives in the urine 12 h after the administration of the dose. The major urinary radioactive product was unmetabolized folic acid.

### DISCUSSION

This study shows that orally administered folic acid is incorporated into the reduced folate pool, and confirms the existence of a breakdown process in man similar to that elucidated in the rat (Pheasant *et al.*, 1981). The control subjects excreted a large proportion of the dose unchanged and, unlike the rat, the excretion of folic acid continued throughout the experiment. The folates present in the urine had a higher $^3H/^{14}C$ ratio than the administered folic acid (see Table IV), indicating that the secondary isotope effect seen in the handling of labelled folic acid by the rat (Connor *et al.*, 1980) also occurs in other species. The same catabolites were identified in the urine of man and rat, but the order of appearance of the two major tritiated species was reversed. If the radioactivity not recovered in the urine or faeces is retained in the tissues, man excretes a higher percentage of the tissue folates as scission products than the rat (Table IV). This is consistent with the suggestion that catabolism proceeds *via* oxidative cleavage on the C9-N10 bond (Murphy *et al.*, 1976; Saleh *et al.*, 1981 since the hepatic NAD/NADH ratio is higher in man than rat (R. A. Harris, personal communication).

Patients with malignant disease excreted less radioactivity in the urine. Intestinal malabsorption of folate is not usually associated with neoplasia (Chanarin, 1979) and, by analogy with the rat, the remaining radioactivity is presumably taken up into tumour tissue (Saleh *et al.*, 1981). This is supported by the inverse correlation between the approximate tumour mass and urinary radioactivity. These patients also incorporated more of the administered folic acid into the reduced folate pool, as shown by the decreased excretion of folic acid and the reduced folic acid/5MeTHF ratio (Table IV), changes which all become more pronounced in the more advanced cases. These results reflect the increased requirement for folate in malignant disease, arising from presence of an additional cell mass.

Despite the increased incorporation of the labelled folate into the reduced folate pool, lower levels of labelled p-acetamidobenzoyl-L-glutamate, the catabolite of the folate polyglutamates, were excreted in the urine (Table IV). This apparent decrease in the catabolism of tissue folate in the presence of a tumour has also been observed in rats, and it has been suggested that it is due to the anoxia of solid tumours, and to the more reducing conditions prevailing in the cytosol of tumour cells (Saleh *et al.*, 1981).

Administered 10-formylfolate (5 mg) was excreted largely unchanged. Approximately 1% of the dose was excreted as 5MeTHF, and a small proportion was present as catabolites (Table VI). This indicates very slow incorporation of 10-formylfolate into the reduced folate pool. The presence of folic acid in the urine suggests that deformylation may precede reduction. Indeed, the folic acid/5MeTHF ratio was similar to that after the administration of folic acid to control subjects. By analogy with folic acid, some of the dose may have been excreted in the faeces and possibly a small percentage retained in the body. The retained radioactivity may reflect the metabolism of that proportion of the dose that was deformylated to folic acid. Thus it appears that in contrast to the rat, 10-formylfolate is utilized only poorly by man, and is probably not reduced to any significant extent in the human body.

Decreasing the dose of folic acid to 1·0 mg or 0·5 mg has been shown to lead to lower recovery of radioactivity in urine, with a greater fall in excretion of folic acid than of the other metabolites (Saleh *et al.*, 1980). In this study a dose about equal to the daily intake of folate was given (60 $\mu$g) and the results confirmed the observations of Pheasant *et al.* (1979). At this low dose, little or no folate was excreted and the only detectable metabolites were scission products. The low recovery of radioactivity in the urine probably indicates that the renal threshold for folates (Johns *et al.*, 1961) has not been exceeded, whereas the catabolites appear to have much lower renal thresholds.

The pattern of urinary metabolites altered dramatically when the same low dose of radiolabelled folic acid was given along with 10-formylfolate (5 mg). A substantial proportion of the folic acid was rapidly excreted unchanged, and relatively little 5MeTHF or scission products were formed. 10-Formylfolate is known to inhibit the reduction of folic acid and dihydrofolate by mammalian dihydrofolate reductase (E.C. 1.5.1.3) *in vitro* (Bertino *et al.*, 1965; Friedkin *et al.*, 1975; A. Sahota, personal communication). These observations suggest that 10-formylfolate also effectively blocks the reduction of folic acid *in vivo*. Hence 10-

formylfolate present in the diet, or formed in the body by the oxidation of 10-formyltetrahydrofolate, could have a significant effect on the activity of dihydrofolate reductase.

This work was carried out with the permission of the ethical committees of the Central Birmingham Health District and the University of Aston in Birmingham.

We are grateful to the Royal Society and the Cancer Research Campaign for financial support.

### REFERENCES

BAKER, B. R., SANTI, D. V., ALMAULA, P. I. & WERKHEISER, W. C. (1964) Analogs of tetrahydrofolic acid X. Synthetic and enzymic studies on the contribution of the p-aminobenzoyl-L-glutamate moiety of pyrimidyl analogs to binding some folic cofactor area enzymes. *J. Med. Chem.*, **7**, 24.

BARFORD, P. A., STAFF, F. J. & BLAIR, J. A. (1977) Retained folates in the rat. *Biochem. J.*, **164**, 601.

BARFORD, P. A., STAFF, F. J. & BLAIR, J. A. (1978) The metabolic fate of (2-$^{14}$C) folic acid and a mixture of (2-$^{14}$C) and (3′, 5′, 7, 9)-$^{3}$H folic acid in the rat. *Biochem. J.*, **174**, 579.

BERTINO, J. R., PERKINS, J. P. & JOHNS, D. G. (1965) Purification and properties of dihydrofolate reductase from Ehrlich Ascites carcinoma cells. *Biochemistry*, **4**, 839.

BLAKLEY, R. L (1959) The reaction of tetrahydropteroyl-L-glutamic acid and related hydropteridines with formaldehyde. *Biochem. J.*, **72**, 707.

BLAKLEY, R. L. (1969) In *The Biochemistry of Folic Acid and Related Compounds*. New York: John Wiley. p. 425.

BUTTERWORTH, C. E., JR, SANTINI, R., JR. & FROMMEYER, W. B. J. (1963) Pteroylglutamate components of American diets as determined by chromatographic fractionation. *J. Clin. Invest.*, **42**, 1929.

CHANARIN, I. (1979) In *The Megaloblastic Anaemias*. 2nd Ed. Oxford: Blackwell. p. 540.

CHANARIN, I. & MCLEAN, A. (1967) Origin of serum and urinary methyltetrahydrofolate in man: some observations on the methylfolate block hypothesis in Addinsonian pernicious anaemia. *Clin. Sci.*, **32**, 57.

CONNOR, M. J., PHEASANT, A. E. & BLAIR, J. A. (1979) The identification of p-acetamidobenzoate as a folate degradation product in rat urine. *Biochem. J.*, **178**, 795.

CONNOR, M. J., BLAIR, J. A. & SAID, H. (1980) Secondary isotope effects in studies using radiolabelled folate tracers. *Nature*, **287**, 253.

FRIEDKIN, M., PLANTE, L. T., CRAWFORD, E. J. & CRUMM, M. (1975) Inhibition of thymidylate synthetase and dihydrofolate reductase by naturally occurring oligoglutamate derivatives of folic acid. *J. biol. Chem.*, **250**, 5614.

JOHNS, D. G., SPERTI, S. & BURGEN, A. S. V. (1961) The metabolism of tritiated folic acid in man. *J. Clin. Invest.*, **40**, 1684.

KRUMDIECK, C. L., FUKUSHIMA, K., FUKUSHIMA, T., SHIOTA, T. & BUTTERWORTH, C. E., JR (1978) A long term study of the excretion of folate and pterins in a human subject after injection of $^{14}$C folic acid, with observations on the effect of diphenylhydantion administration. *Am. J. Clin. Nutr.*, **31**, 88.

MURPHY, M., KEATING, M., BOYLE, P., WEIR, D. G. & SCOTT, J. M. (1976) Elucidation of the mechanism of folate catabolism in the rat. *Biochem. Biophys. Res. Commun.*, **71**, 1017.

PHEASANT, A. E., BLAIR, J. A. & ALLAN, R. N. (1979) Folic acid metabolism in man. *Dev. Biochem.*, **4**, 327.

PHEASANT, A. E., CONNOR, M. J. & BLAIR, J. A. (1981) The metabolism and physiological disposition of radioactively labelled folate derivatives in the rat. *Biochem. Med.* **26**, 435.

RATANASTHIEN, K., BLAIR, J. A., LEEMING, R. J., COOKE, W. T. & MELIKIAN, V. (1974) Folates in human serum. *Clin. Pathol.*, **27**, 875.

RATANASTHIEN, K. (1975) *Metabolism and Handling of Folates in the Mammal Especially Man*. PhD Thesis, University of Aston in Birmingham.

SALEH, A. M., PHEASANT, A. E., BLAIR, J. A. & ALLAN, R. N. (1980) The effect of malignant disease on the metabolism of pteroylglutamic acid in man. *Biochem. Soc. Trans.*, **8**, 566.

SALEH, A. M., PHEASANT, A. E. & BLAIR, J. A. (1981) Folate catabolism in tumour-bearing rats and rats treated with methotrexate. *Br. J. Cancer*, **44**, 700.

SANTINI, R., BREWSTER, B. S. & BUTTERWORTH, C. E., JR (1964) The distribution of folic acid active compounds in individual foods *Am. J. Clin. Nutr.*, **14**, 205.


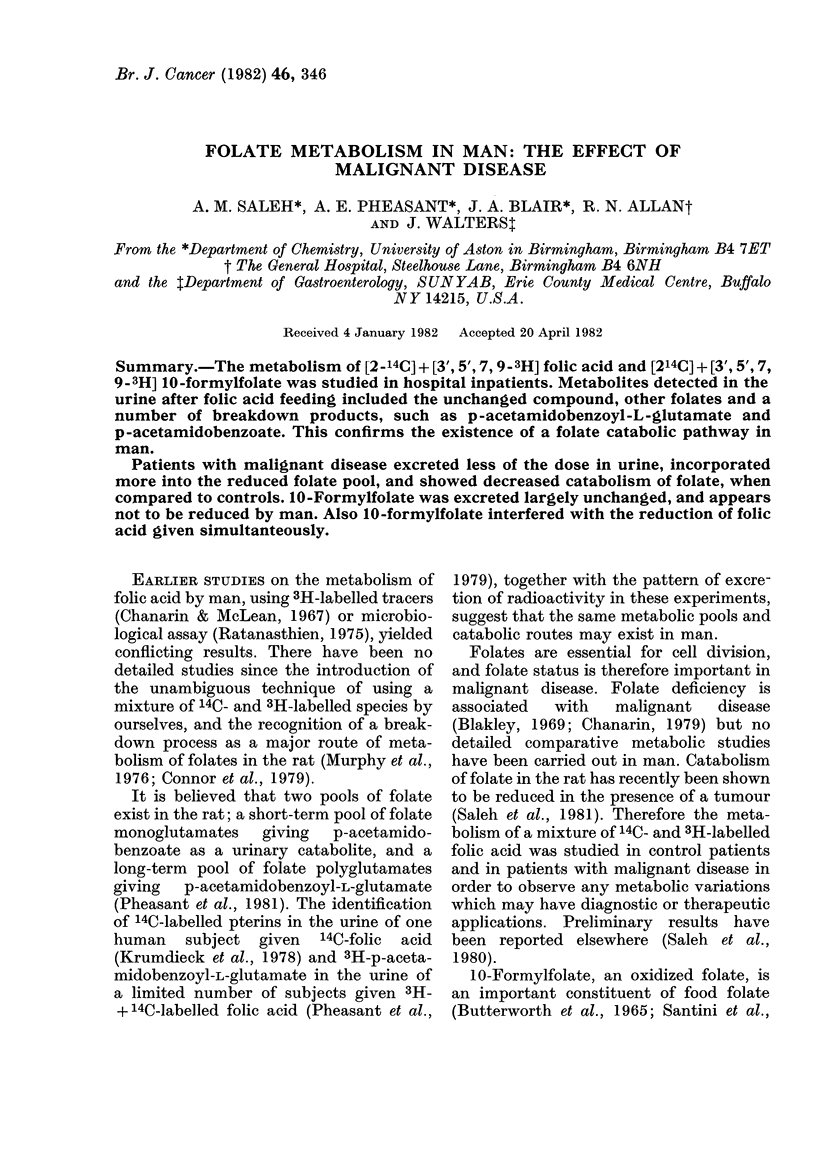

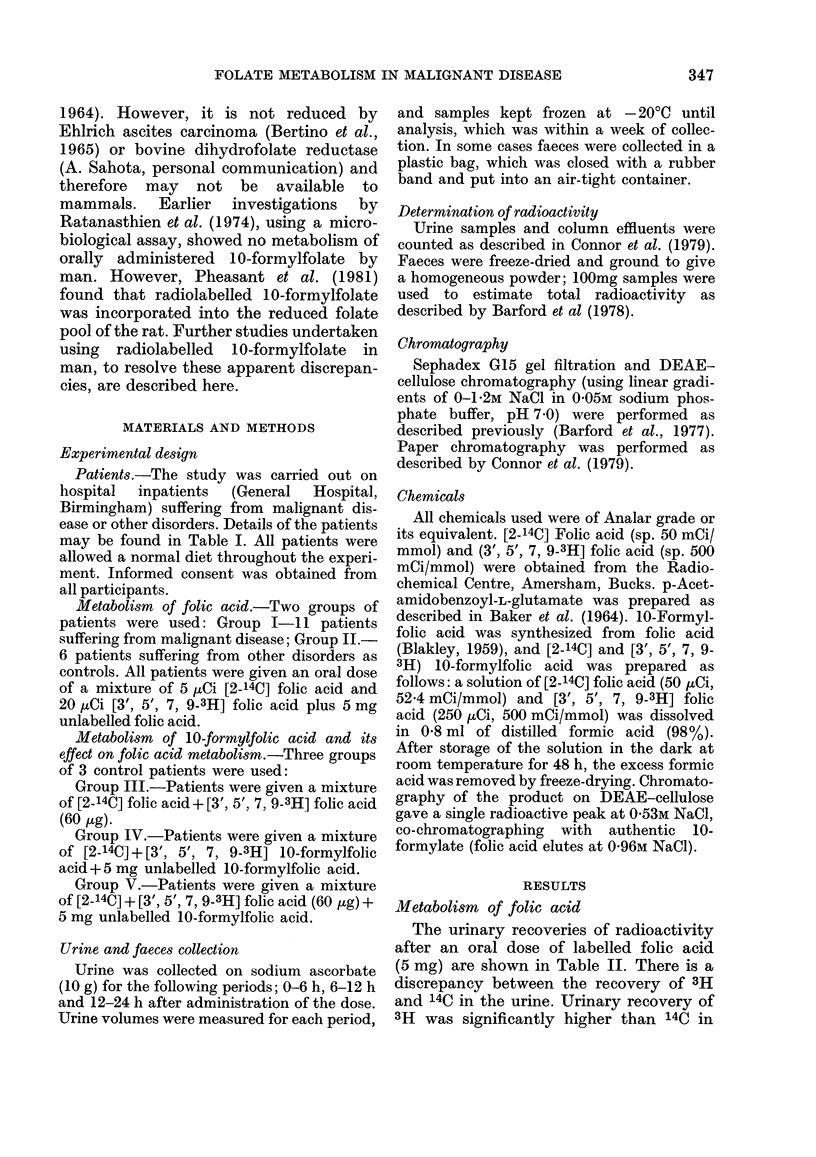

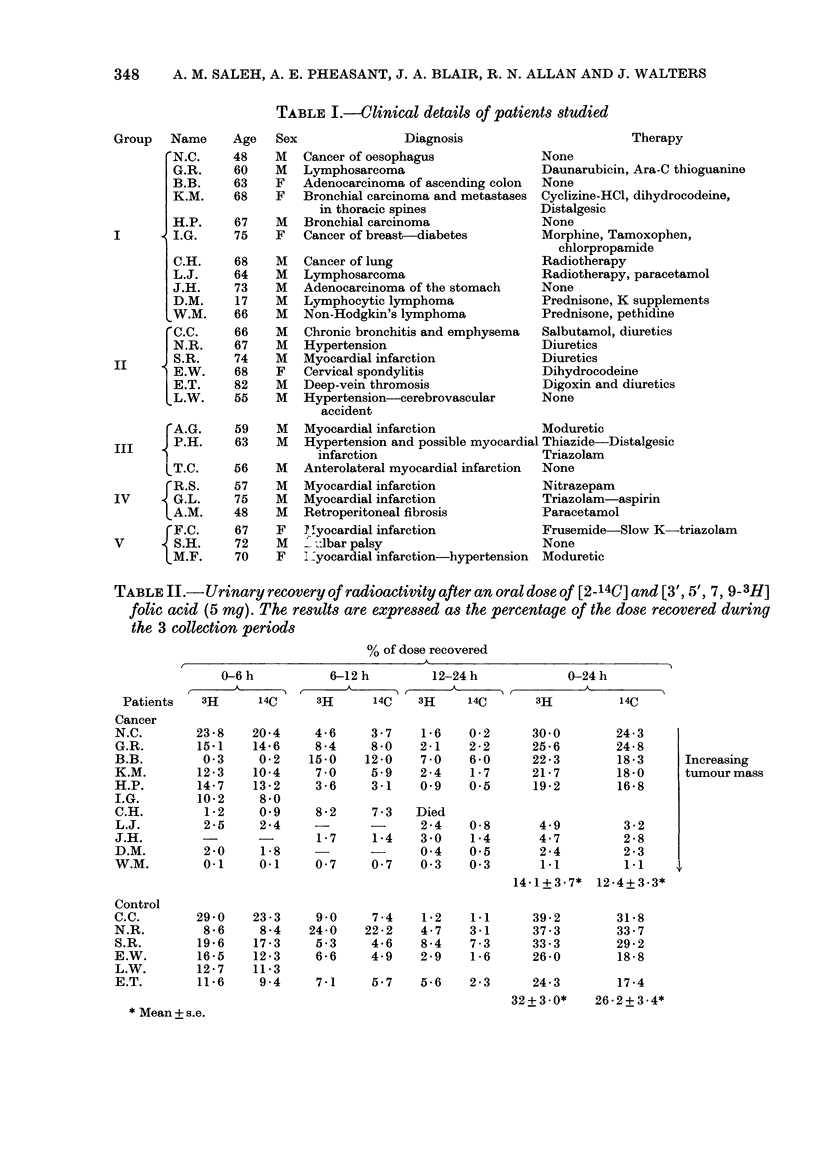

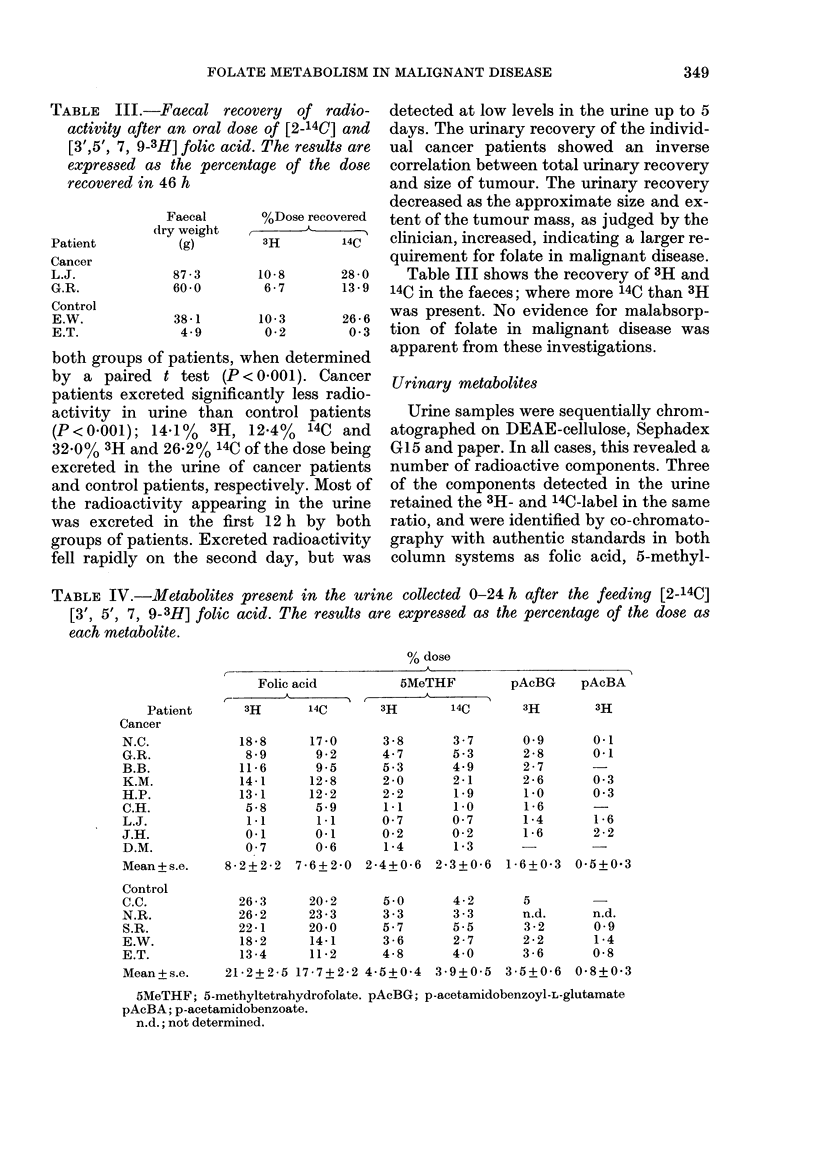

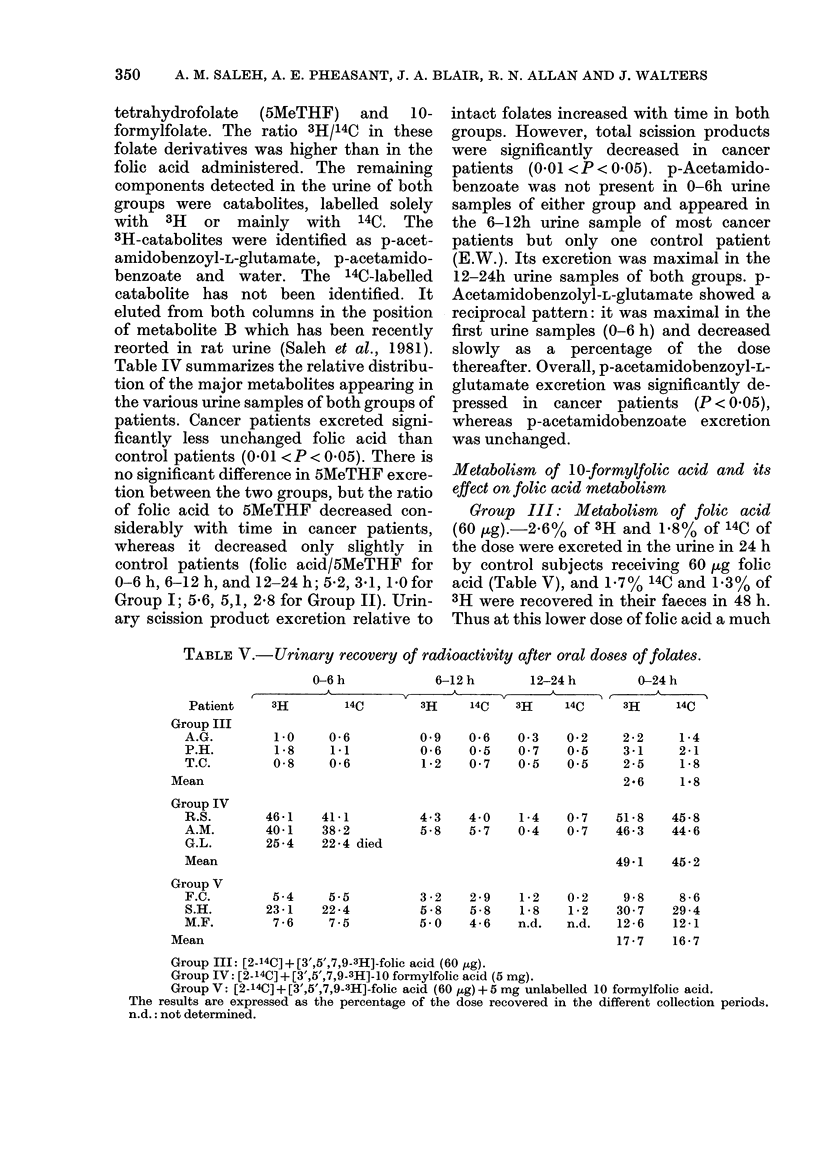

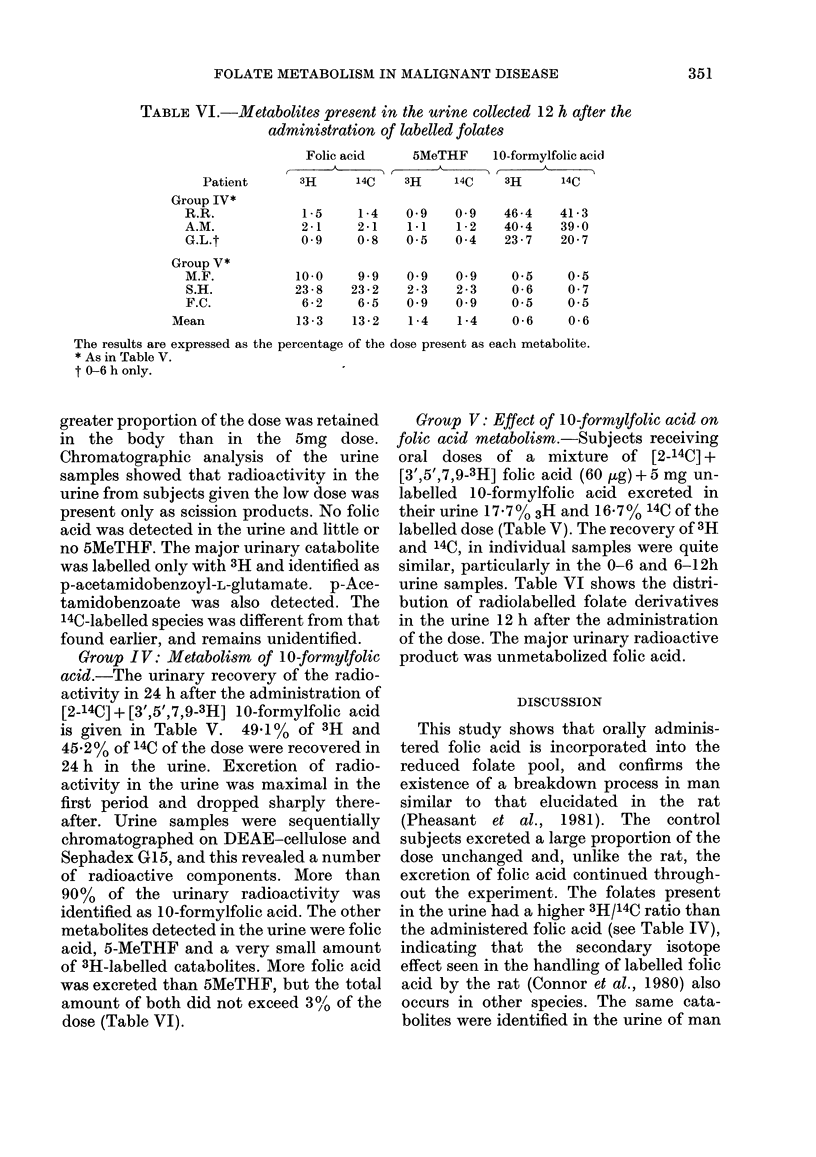

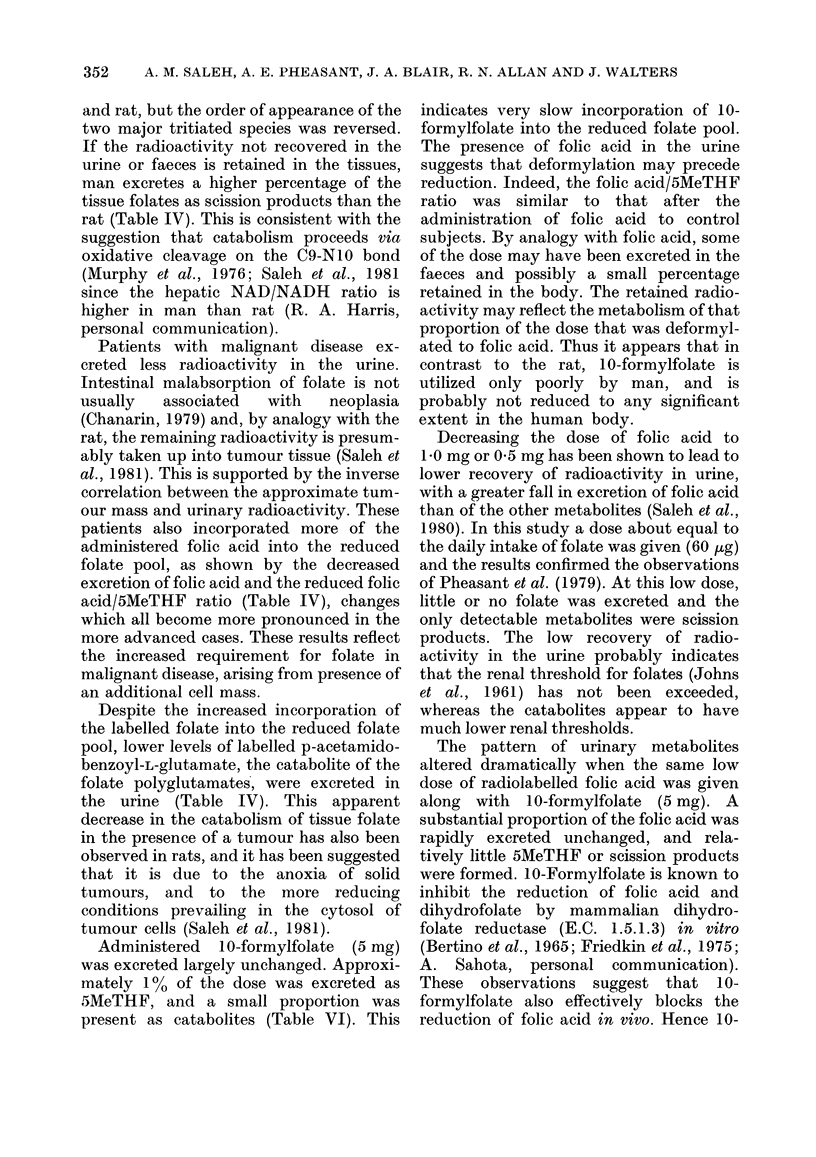

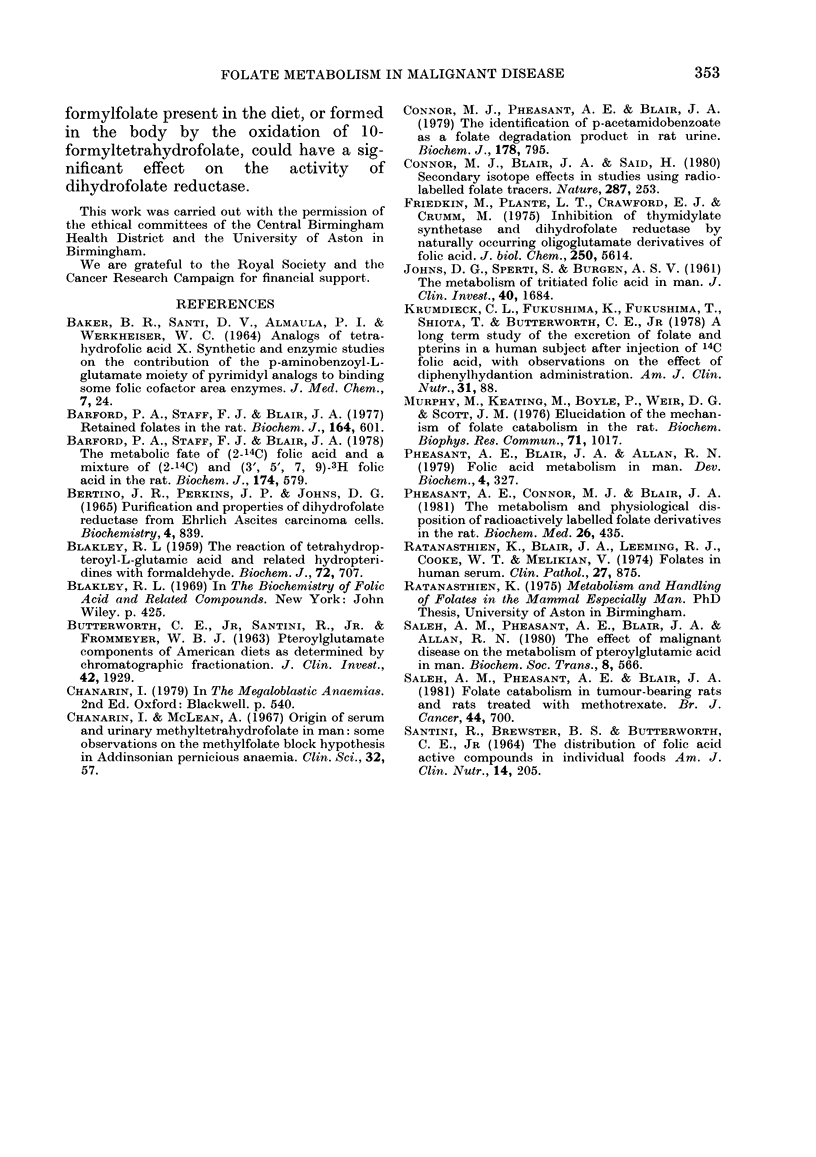


## References

[OCR_01046] BAKER B. R., SANTI D. V., ALMAULA P. I., WERKEISER W. C. (1964). ANALOGS OF TETRAHYDROFOLIC ACID. X. SYNTHETIC AND ENZYMIC STUDIES ON THE CONTRIBUTION OF THE P-AMINOBENZOYL-L-GLUTAMATE MOIETY OF PYRIMIDYL ANALOGS TO BINDING TO SOME FOLIC COFACTOR AREA ENZYMES.. J Med Chem.

[OCR_01064] BERTINO J. R., PERKINS J. P., JOHNS D. G. (1965). PURIFICATION AND PROPERTIES OF DIHYDROFOLATE REDUCTASE FROM EHRLICH ASCITES CARCINOMA CELLS.. Biochemistry.

[OCR_01070] BLAKLEY R. L. (1959). The reaction of tetrahydropteroylglutamic acid and related hydropteridines with formaldehyde.. Biochem J.

[OCR_01080] BUTTERWORTH C. E., SANTINI R., FROMMEYER W. B. (1963). THE PTEROYLGLUTAMATE COMPONENTS OF AMERICAN DIETS AS DETERMINED BY CHROMATOGRAPHIC FRACTIONATION.. J Clin Invest.

[OCR_01058] Barford P. A., Staff F. J., Blair J. A. (1978). The metabolic fate of (2-14C)folic acid and a mixture of (2-14C)- and (3',5',9-3h)-folic acid in the rat.. Biochem J.

[OCR_01055] Barford P. A., Staff R. J., Blair J. A. (1977). Retained folates in the rat.. Biochem J.

[OCR_01091] Chanarin I., McLean A. (1967). Origin of serum and urinary methyltetrahydrofolate in man. Some observations on the methylfolate block hypothesis in Addisonian pernicious anaemia.. Clin Sci.

[OCR_01104] Connor M. J., Blair J. A., Said H. (1980). Secondary isotope effects in studies using radiolabelled folate tracers.. Nature.

[OCR_01098] Connor M. J., Pheasant A. E., Blair J. A. (1979). The identification of p-acetamidobenzoate as a folate degradation product in rat urine.. Biochem J.

[OCR_01109] Friedkin M., Plante L. T., Crawford E. J., Crumm M. (1975). Inhibition of thymidylate synthetase and dihydrofolate reductase by naturally occurring oligoglutamate derivatives of folic acid.. J Biol Chem.

[OCR_01116] JOHNS D. G., SPERTI S., BURGEN A. S. (1961). The metabolism of tritiated folic acid in man.. J Clin Invest.

[OCR_01121] Krumdieck C. L., Fukushima K., Fukushima T., Shiota T., Butterworth C. E. (1978). A long-term study of the excretion of folate and pterins in a human subject after ingestion of 14C folic acid, with observations on the effect of diphenylhydantoin administration.. Am J Clin Nutr.

[OCR_01130] Murphy M., Keating M., Boyle P., Weir D. G., Scott J. M. (1976). The elucidation of the mechanism of folate catabolism in the rat.. Biochem Biophys Res Commun.

[OCR_01141] Pheasant A. E., Connor M. J., Blair J. A. (1981). The metabolism and physiological disposition of radioactively labelled folate derivatives in the rat.. Biochem Med.

[OCR_01147] Ratanasthien K., Blair J. A., Leeming R. J., Cooke W. T., Melikian V. (1974). Folates in human serum.. J Clin Pathol.

[OCR_01169] SANTINI R., BREWSTER C., BUTTERWORTH C. E. (1964). THE DISTRIBUTION OF FOLIC ACID ACTIVE COMPOUNDS IN INDIVIDUAL FOODS.. Am J Clin Nutr.

[OCR_01157] Saleh A. M., Pheasant A. E., Blair J. A., Allan R. N. (1980). The effect of malignant disease on the metabolism of pteroylglutamic acid in man.. Biochem Soc Trans.

[OCR_01163] Saleh A. M., Pheasant A. E., Blair J. A. (1981). Folate catabolism in tumour-bearing rats and rats treated with methotrexate.. Br J Cancer.

